# Relaxation-Based Radiometric Normalization for Multitemporal Cross-Sensor Satellite Images

**DOI:** 10.3390/s23115150

**Published:** 2023-05-28

**Authors:** Gabriel Yedaya Immanuel Ryadi, Muhammad Aldila Syariz, Chao-Hung Lin

**Affiliations:** 1 Department of Geomatics, National Cheng Kung University, Tainan City 70101, Taiwan; p68117018@gs.ncku.edu.tw (G.Y.I.R.); aldilasyariz@its.ac.id (M.A.S.); 2 Department of Geomatics Engineering, Institut Teknologi Sepuluh Nopember, Surabaya 60111, Indonesia

**Keywords:** multitemporal cross-sensor image, visual consistency, image normalization, IR-MAD, relaxation

## Abstract

Multitemporal cross-sensor imagery is fundamental for the monitoring of the Earth’s surface over time. However, these data often lack visual consistency because of variations in the atmospheric and surface conditions, making it challenging to compare and analyze images. Various image-normalization methods have been proposed to address this issue, such as histogram matching and linear regression using iteratively reweighted multivariate alteration detection (IR-MAD). However, these methods have limitations in their ability to maintain important features and their requirement of reference images, which may not be available or may not adequately represent the target images. To overcome these limitations, a relaxation-based algorithm for satellite-image normalization is proposed. The algorithm iteratively adjusts the radiometric values of images by updating the normalization parameters (slope (α) and intercept (β)) until a desired level of consistency is reached. This method was tested on multitemporal cross-sensor-image datasets and showed significant improvements in radiometric consistency compared to other methods. The proposed relaxation algorithm outperformed IR-MAD and the original images in reducing radiometric inconsistencies, maintaining important features, and improving the accuracy (MAE = 2.3; RMSE = 2.8) and consistency of the surface-reflectance values (R^2^ = 87.56%; Euclidean distance = 2.11; spectral angle mapper = 12.60).

## 1. Introduction

**Figure 1:**
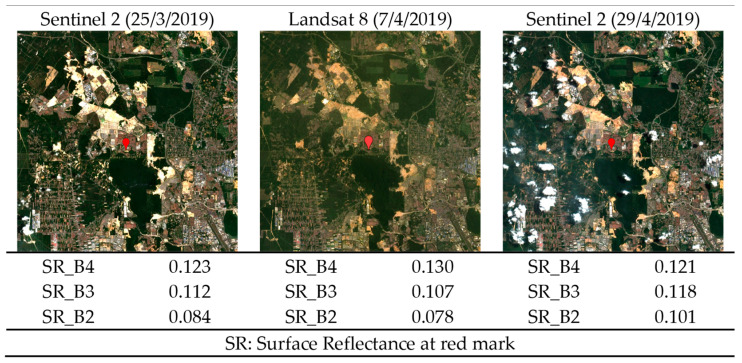
Inconsistency of visual and radiometric values between multitemporal cross-sensor images.

Multitemporal cross-sensor imagery has become increasingly common in remote-sensing applications, as the data obtained allow the analysis of changes and trends over time [1,2]. However, one of the major challenges in the usage of multitemporal cross-sensor imagery is the removal of radiometric inconsistency among images, as shown in Figure 1, which can lead to errors in analysis and the misinterpretation of results [3]. Factors such as different sensor characteristics, topography, atmospheric conditions, and sun-sensor geometry can all contribute to inconsistencies among images [4,5]. To address this issue, researchers have developed various methods for image normalization. Image normalization involves the adjustment of the radiometric values of an image to make it more comparable to the other images in a dataset [6,7]. Histogram matching is a commonly used method for image normalization, in which the histogram of an image is matched to a reference histogram [8]. Another method is dark–bright target normalization, which manually selects dark and bright targets in the reference image and normalizes the target image [4,9]. One of the most widely used methods for image normalization is linear regression using iteratively reweighted multivariate alteration detection (IR-MAD) [10]. The application of IR-MAD involves the detection and correction of the differences between images using statistical techniques; the radiometric values of each image are adjusted iteratively until the differences between the images are minimized [11,12]. Although these methods have shown promising results, they also have limitations. For example, histogram matching can be sensitive to outliers [13], while dark–bright target normalization requires the presence of a suitable target in the image. While effective, IR-MAD requires a reference image and may not perform well in complex datasets with atmospheric or surface conditions, seasonal changes, and other variations.

To address the limitations of current image-normalization methods, this study proposes a novel relaxation algorithm that improves image consistency both quantitatively and qualitatively without requiring a reference image. The relaxation algorithm iteratively adjusts the radiometric values of images using a relaxation parameter that controls the degree of smoothing. By applying an iterative process to reduce radiometric differences and adjust radiometric values, the proposed method aims to minimize errors in multitemporal cross-sensor images. The proposed method was tested on multitemporal cross-sensor-image datasets with complex features, such as seasonal changes, water, topography, desert, snow, and cloud cover, and demonstrated significant improvements in image consistency compared to other methods.

## 2. Data and Methods

### 2.1. Satellite Data

Several multitemporal cross-sensor images acquired from Landsat 8 (LANDSAT/LC08/C02/T1_L2) and Sentinel 2 (COPERNICUS/S2_SR) surface-reflectance products were used to evaluate the proposed relaxation method. These datasets were chosen based on diversity in terms of geographic features and atmospheric conditions. The details of the datasets are summarized in Table 1. Note that all spectral bands of the images except the thermal, cirrus, and panchromatic bands were used.

Dataset #1 consists of surface-reflectance images from Landsat 8 and Sentinel 2 sensors over Tainan City, Taiwan, acquired from January to March 2020. The main objective of this dataset is to analyze the impact of normalization on urban areas and airport images, which could provide valuable information on land-cover and land-use patterns for use in further studies, such as urbanization studies [14,15]. Dataset #2 comprises surface-reflectance images of Mut, Egypt, one of the ancient cities located in the Western Desert. The city experiences a hot and arid climate, with little rainfall. The landscape is dominated by sand dunes and rocky mountains. This dataset focuses on a specific part of the city, and its objective is to analyze the impact of normalization on desert images, which poses significant challenges due to atmospheric aerosols and bright-reflective surfaces [16,17]. Dataset #3 includes surface-reflectance images of Yakutsk, Russia, a city in the Sakha Republic known for its extremely cold subarctic winters. These images were acquired between February and March 2021, at the end of the winter season. The primary objective of this dataset is to investigate the impact of normalization on snowy images, which can be challenging to process due to their high reflectance, which often results in loss of detail and information in bright areas [18].

Datasets #4 and #5 are composed of surface-reflectance images of urban areas in Manaus, Brazil, and Dubbo, Australia, respectively. Both datasets contain cloudy images, but the amount of cloud pixels differs between the two. Dataset #4 has fewer cloud pixels than Dataset #5. The objective of these datasets is to study the impact of normalization on images with cloud pixels. Clouds can present challenging issues in image processing as they can obscure the underlying surface features and vary in thickness and height, resulting in different levels of reflectance [19,20]. Dataset #6 comprises surface-reflectance images from Legrena, a small coastal city located in southeastern Greece. The city’s landscape is characterized by rocky cliffs, valleys, and beaches, making it a unique dataset with diverse geographical features. The dataset was selected for its water bodies, the topography effects caused by cliffs, and shadow effects on the acquired images. Processing of images of water bodies is challenging due to their significantly low surface-reflectance values compared with other inland surfaces [21]. Water is influenced by various factors, such as atmospheric conditions, water depth, and the sun angle, which can result in varying levels of surface reflectance and color [22]. Moreover, the topography of a region can lead to variations in surface reflectance due to changes in slope, aspect, and terrain ruggedness [23,24]. Additionally, shadow and shading can affect images, making accurate analysis difficult [25]. The objective of this dataset is to analyze the impact of normalization on these areas. Dataset #7 is the final dataset, and it consists of surface-reflectance images over Nashville City, Tennessee, USA. The images were acquired during the transition from summer to autumn season. The objective of this dataset is to identify the impact of normalization on seasonally affected pixels. Seasonally affected pixels are pixels in an image that change due to seasonal environmental changes, such as variations in temperature, humidity, and atmospheric conditions [26,27].

### 2.2. Overview of Relaxation Method

**Figure 2:**
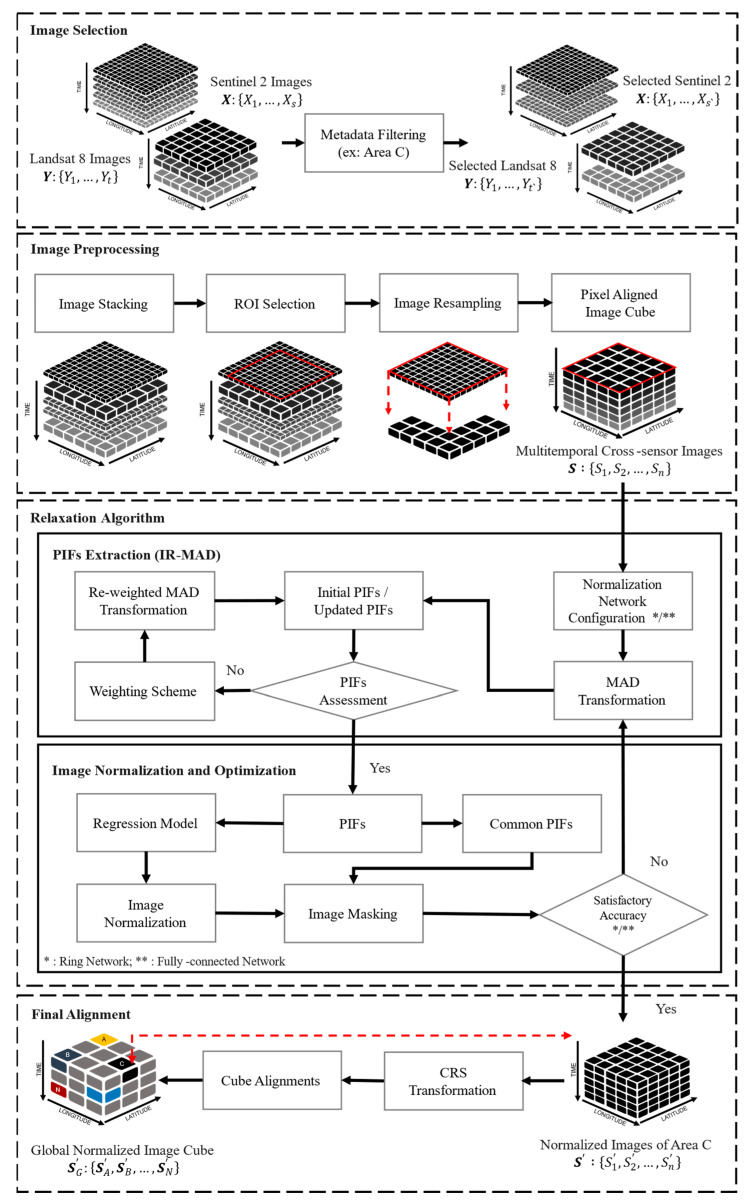
Workflow of image normalization with relaxation algorithm.

The proposed relaxation method consists of five steps, as illustrated in Figure 2. The first step involves selecting images from two satellite datasets, followed by image pre-processing in the second step. In the third step, pseudo-invariant feature (PIF) extraction is carried out using IR-MAD between paired images. Image normalization and optimization are performed using a regression model and satisfactory accuracy assessment in the next step. The third and fourth steps are repeated iteratively to improve the surface-reflectance consistency and enhance image visualization. Finally, the normalized images are aligned locally and globally to create a global normalized image cube. Each of these steps is described in detail below.

#### 2.2.1. Image Selection and Preprocessing

Metadata filtering is an initial step in data processing, in which unsuitable images are filtered out. The parameters in this filtering process involve metadata of images, such as sensor type, acquisition date, and geographical location. Dates are filtered to obtain images acquired during specific seasons or events, while geographic filtering is useful in selection of images of a particular area of interest. Row- and path-based filtering are important for selecting images with similar viewing angles or creating mosaics. The objective of this filtering is to ensure that the selected images meet the specific analysis requirements and yield dependable results.

In this study, image pre-processing was used to prepare the satellite images for downstream analysis. The initial step in image pre-processing is image stacking, where Sentinel 2 images are combined with Landsat 8 images to create a seamless composite image [28,29]. Next, regions of interest (ROIs) are selected to ensure that the images have the same area and viewpoint of objects. This step is essential to eliminate any distortions caused by differences in sensor viewing angles and ensure that the images are correctly aligned [30]. Image resampling is then applied, whereby the resolution of Sentinel 2 images is reduced to a 30-m spatial resolution. This step is necessary to ensure compatibility with Landsat 8 images, which have a lower spatial resolution. Finally, the pre-processing stage is concluded with a pixel-aligned image cube, in which the images are aligned based on their pixels.

#### 2.2.2. PIF Extraction

The next step in the image-processing pipeline is the extraction of pseudo-invariant features (PIFs). These PIFs refer to stable features on the Earth’s surface that remain relatively unchanged over time, and they can be used to normalize remote-sensing data [31]. Examples of PIFs include urban areas, roads, and bare soil, which tend to have consistent surface-reflectance values over time. Although PIFs are not completely invariant and can be affected by seasonal or weather-related changes, they remain relatively stable and can be used to account for variations in atmospheric and environmental conditions during image normalization [32]. By incorporating PIFs into the normalization process, the accuracy and comparability of remote-sensing data can be improved, making it easier to analyze and interpret changes over time.

In this study, we performed iteratively reweighted multivariate alteration detection (IR-MAD) to select the PIFs from paired images. The IR-MAD approach is an efficient method to extract time-invariant features for radiometric normalization that builds upon the traditional multivariate alteration detection (MAD) algorithm [33,34]. As shown in Figure 2, the input dataset for PIF extraction is an image cube of multi-temporal and cross-sensor images denoted as $S:\left\{S_{1},S_{2},\ldots ,S_{n}\right\}$, where n refers to the number of images. This image cube is the result of the image-pre-processing stage, in which the images from the two sensors are stacked, resampled and pixel aligned, so they are compatible with each other for further processing. The MAD is a method used to identify changes between two multispectral images, which utilizes traditional canonical correlation analysis (CCA) to model the linear combination of two multispectral images based on their order of correlation [35]. The differences between ordered pairs are referred to as MAD variates, which are represented by Equation (1). These MAD variates illustrate the variance of the two multispectral images ($S_{1}$ and $S_{n}$) based on their total *k*-spectral bands, ranked from the highest to the lowest. The eigenvectors **a** and **b**, along with their corresponding eigenvalues, are given in Equation (2). In this equation, $C_{11}$ and $C_{nn}$ represent the variance matrix of a single set variable ($S_{1}$ and $S_{n}$, respectively), and the covariance between them is represented by $C_{1n}$ and $C_{n1}$.

(1) $$\left[\begin{array}{c} {MAD}_{1}\\ \begin{array}{c} {MAD}_{2}\\ {MAD}_{3}\\ \vdots \end{array}\\ {MAD}_{k} \end{array}\right]=\left[\begin{array}{c} a_{k}^{T}S_{1}-b_{k}^{T}S_{n}\\ \begin{array}{c} a_{k-1}^{T}S_{1}-b_{k-1}^{T}S_{n}\\ a_{k-2}^{T}S_{1}-b_{k-2}^{T}S_{n}\\ \vdots \end{array}\\ a_{1}^{T}S_{1}-b_{1}^{T}S_{n} \end{array}\right],$$

(2) $$C_{1n}{C_{nn}}^{-1}C_{n1}a=\rho^{2}C_{11}a;C_{n1}{C_{11}}^{-1}C_{1n}b=\rho^{2}C_{nn}b.$$

When the difference image follows a multivariate normal distribution, the sum of squared MAD variates (Equation (3)) can be shown to follow a chi-square distribution $X^{2}$ with f degrees of freedom equal to the number of spectral bands, expressed as Equation (4).

(3) $$G=\sum_{i=1}^{k}{\left(\frac{{MAD}_{i}}{\sigma_{{MAD}_{i}}}\right)}^{2},$$

(4) $$P_{X^{2},f}(G)=\sum_{i=1}^{f}\left(G)\in X^{2}(f\right),$$

where $\sigma_{{MAD}_{i}}$ represents the standard deviations of MAD variates and $\left\{{MAD}_{1},\cdots ,{MAD}_{k}\right\}$ is defined as $\sigma_{{MAD}_{i}}=\sqrt{\sum_{i=1}^{k}{MAD}_{i}-\overset{-}{{MAD}_{i}}/k}$. The resulting distribution can be used to identify statistically significant anomalies in the difference image. This is achieved by calculating the probability that the sum of squared MAD variates will exceed a certain threshold within the chi-square distribution [33,36]. The pixels that satisfy the following probability conditions are selected as PIFs candidates.

(5) $$\mathrm{Pr}\left(no\;change\right)=1-P_{X^{2},f}(G),$$

where Pr⁡(no change) is used to select the PIFs in kernel space. The selection of PIFs is formulated as

(6) $$\omega_{j}=PIFs=P\left\{\mathrm{Pr}\left(no\;change\right)>t\right\}≅P\left\{X^{2}\left(f\right)>t\right\},$$

where *t* represents the fixed threshold from percentiles in the $X^{2}$ distribution, usually larger than 0.9 to mask out the water pixels, cloud pixels, and shadow pixels in the image [33,37]. Next, an iterative reweighting scheme is performed to improve the detection of weak anomalies by using $\omega_{j}$ as a weight factor for selected pixel-*j*. The reweighting scheme involves estimating weights based on the PIF selection ($\omega_{j}$) in the previous iteration, which helps to identify weak anomalies that may have been missed in the initial weighting scheme ($\omega_{j}=1$) [38]. This weight enters the calculation of mean, variance, and covariances (*N* is the number of pixels) of $S_{1}$ and $S_{n}$, respectively.

(7) $$\overset{-}{S_{1}}=\frac{\sum_{j=1}^{N}\omega_{j}S_{j1}}{\sum_{j=1}^{N}\omega_{j}};\overset{-}{S_{n}}=\frac{\sum_{j=1}^{N}\omega_{j}S_{jn}}{\sum_{j=1}^{N}\omega_{j}},$$

for the mean value of $S_{1}$ and $S_{n}$, and

(8) $$C_{11}=\frac{\sum_{j=1}^{N}\omega_{j}{\left(S_{j1}-\overset{-}{S_{1}}\right)}^{2}}{(N-1)\sum_{j=1}^{N}\omega_{j}/N};C_{nn}=\frac{\sum_{j=1}^{N}\omega_{j}{\left(S_{jn}-\overset{-}{S_{n}}\right)}^{2}}{(N-1)\sum_{j=1}^{N}\omega_{j}/N};C_{1n}=\frac{\sum_{j=1}^{N}\omega_{j}(S_{j1}-\overset{-}{S_{1}})(S_{jn}-\overset{-}{S_{n}})}{(N-1)\sum_{j=1}^{N}\omega_{j}/N},$$

for the variance and covariance between $S_{1}$ and $S_{n}$.

In IR-MAD, iterations are performed until a convergence criterion is reached, which is usually determined by a maximum number of iterations or a minimum change in the MAD score between iterations. The MAD score measures the magnitude of change in pixel values between two images and is updated in each iteration. Once the MAD score reaches a stable value, the iteration is stopped, and PIFs are extracted.

#### 2.2.3. Image Normalization and Optimization

After the PIFs are selected and extracted, the following step is image normalization. In this study, we applied the regression model (Equation (9)) to transform the radiometric condition of the target image (image $S_{n}$) into the radiometric condition of the reference image (image $S_{1}$) [39,40,41]. This regression utilizes PIFs from previous stage to remove the pixels that are constantly changing, such as clouds, water, and even vegetation in some cases.

(9) $$S_{n}^{\prime }=\alpha^{n\to 1}\left(S_{n}\right)+\beta^{n\to 1},$$

where $\alpha^{n\to 1}$ and $\beta^{n\to 1}$ are the slope and intercept of image $S_{n}$ to image $S_{1}$, which is obtained from Equation (10), below. In this equation, $\sigma ({PIF}_{S1})$ and $\sigma ({PIF}_{Sn})$ denote the standard-deviation values from PIF images of $S_{1}$ and $S_{n}$. Next, $\bar{{PIF}_{S1}}$ and $\bar{{PIF}_{Sn}}$ represent the mean values of PIF images of $S_{1}$ and $S_{n}$ respectively.

(10) $$\alpha^{n\to 1}=\frac{\sigma ({PIF}_{S1})}{\sigma ({PIF}_{Sn})};\;\beta^{n\to 1}=\bar{{PIF}_{S1}}-\alpha^{n\to 1}\cdot \bar{{PIF}_{Sn}}.$$

Furthermore, we propose an iterative optimization algorithm to obtain consistent surface-reflectance values of normalized images. The proposed relaxation algorithm minimizes a real value function or an error function by constructing a sequence of iterations [42,43]. The algorithm operates by starting with an initial value of normalization parameters (slope (α) and intercept (β) variables) from the previous normalization result and repeatedly updates them until the desired level of convergence is obtained. The proposed algorithm gradually aligns the radiometric values of the target image with those of any reference image, which results in a more consistent set of images.

**Figure 3:**
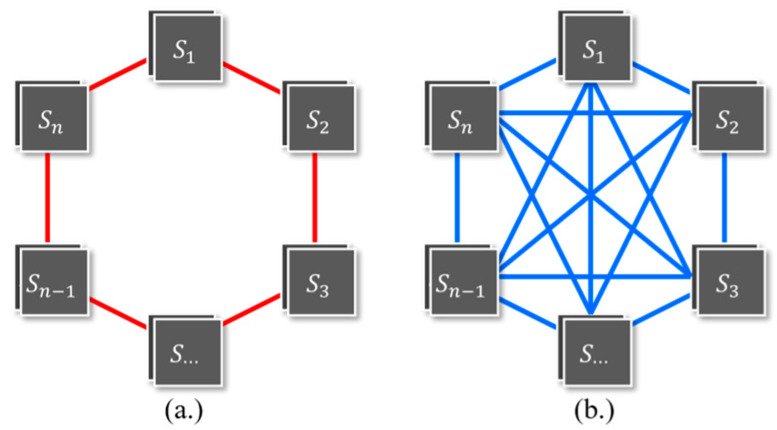
Normalization-network configuration. (**a**) Ring network, with each image linked to two neighboring images. (**b**) Fully connected network, with each image linked to all images in dataset.

In this study, two different networks were used to perform relaxation for image normalization, as illustrated in Figure 3. These two networks were used to align the radiometric conditions of all images with each other without relying on a reference image. Figure 3a shows a network in which each of the *n*-images in the dataset is connected to its two neighboring images (*n* − 1 and *n* + 1), and the first image is connected to the last image in the dataset. The network in this figure is called a ring network. In contrast, Figure 3b displays more links between its images than Figure 3a, with each image in this network connected to all the other images. This network is called a fully connected network. The inputs to these networks are multitemporal cross-sensor images, and the outputs are the normalized images. Hence, two different error functions were used in this study to match the conditions in Figure 3, expressed in Equations (11) and (12).

(11) $$\underset{A,B}{\min}E(O_{1},O_{2},\ldots ,O_{n})=\frac{\sum_{i=1}^{n}O_{i}-\sum_{i=1}^{n}\left\{\begin{array}{c} O\left(A^{\left(i-1\right)\to i}S_{\left(i-1\right)}+B^{\left(i-1\right)\to i}\right)+\\ O(A^{(i+1)\to i}S_{\left(i+1\right)}+B^{(i+1)\to i}) \end{array}\right\}}{2},$$

(12) $$\underset{A,B}{\min}E\left(O_{1},O_{2},\ldots ,O_{n}\right)=\frac{\sum_{i=1}^{n}O_{i}-\sum_{i=1}^{n}\left\{O\left(A^{i\to n}S_{i}+B^{i\to n}\right)\right\}}{2}.$$

In the previous equations, $A=\left\{\alpha^{1\to 2};\alpha^{1\to 3};\ldots ;\alpha^{(n-1)\to n}\right\}$ and $B=\left\{\beta^{1\to 2};\beta^{1\to 3};\ldots ;\beta^{(n-1)\to n}\right\}$ were the set of slope and intercept components of the normalization. These sets (A,B) are updated through a relaxation-iteration process until the minimum error value is obtained. Next, $O:\{O_{1},O_{2},\ldots ,O_{n}\}$ represents the PIFs masked images formulated in Equations (13) and (14). These are masked images that are generated using common PIFs, which are selected PIFs that are shared among multiple images and can be obtained by combining multiple PIF-selection results.

(13) $$PIFs\left(common\right)={\left(PIFs\right)}_{1}*{\left(PIFs\right)}_{\ldots }*{\left(PIFs\right)}_{n},$$

(14) $$O_{1}=S_{1}*PIFs\left(common\right);O_{\cdots }=S_{\cdots }*PIFs\left(common\right);O_{n}=S_{n}*PIFs\left(common\right)$$

The $E(O_{1},O_{2},\ldots ,O_{n})$ from Equations (11) and (12) are error functions in this study, with the objective of obtaining the minimum total error value of each image against other images in the dataset. Specifically, Equation (11) is the error function for the relaxation algorithm, which is carried out by the ring network (Figure 3a). This equation calculates the sum error from each PIF-masked image with respect to their two neighboring images. Equation (12) is the error function for the relaxation algorithm, which is performed by using a fully connected network (Figure 3b). This equation calculates the total error from each PIF-masked image against all its neighboring images.

The name of relaxation is derived from the fact that it gradually “relaxes” the constraints on the normalization components, allowing them to gradually approach a solution that satisfies all the constraints simultaneously. This process is repeated until the error function is minimized to an acceptable level, at which point the final set of values can be considered consistent.

#### 2.2.4. Image-Cube Creations and Alignments

The creation of an image cube involves stacking multiple images acquired at different times to form a three-dimensional data structure. The image cube in this study is the result of the normalization process and represents a time series of images with consistent radiometric values [44,45]. It is created by selecting the best result of normalization from several iterations and conducting a satisfactory accuracy assessment.

After the normalized images are selected, they are aligned to create a local cube with the times of acquisition in ascending order, along with their specific locations. This alignment is critical to ensure that the images are in the correct spatial orientation relative to each other, allowing accurate comparison and analysis over time [46]. After the local cube is created, a coordinate-reference-system transformation is performed to make a global image cube that contains local image cubes. The global-coordinate-reference system used for the image cube is typically WGS 84, which is a commonly used global reference system in remote sensing. This transformation is necessary to ensure that images are accurately georeferenced, enabling accurate spatial analysis and interpretation [47]. The resulting image cube can be used to detect and analyze changes over time, such as land-use and land-cover changes, vegetation dynamics, and urban growth.

## 3. Experimental Results and Discussion

### 3.1. Qualitative Assessment

During the visual assessment stage, we compared five out of seven datasets separately, which consisted of images with seasonal changes (United States of America), as well as water and topography (Greece), desert (Egypt), snow (Russia), and cloud (Brazil) features. The remaining datasets (Taiwan and Australia) are shown in Appendix A. This step allowed us to evaluate the effectiveness of our normalization algorithm in preserving visual quality while reducing spectral variability.

#### 3.1.1. Seasonal Features

**Figure 4:**
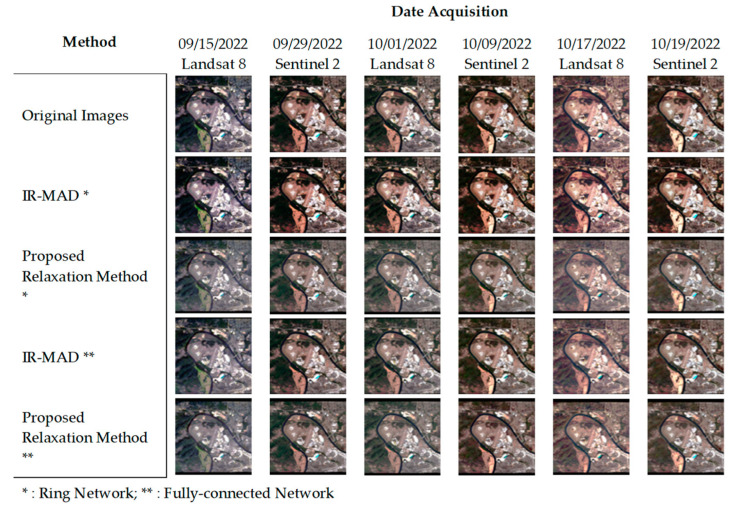
Comparison of normalization results on seasonal-features images, USA image dataset.

The image dataset for the United States of America image (Figure 4) presented a challenging task due to the seasonal changes that affected the urban areas. However, all the normalization methods, including our proposed relaxation algorithm, performed well in reducing the image inconsistencies. However, our approach stood out as it improved the image consistency and maintained the seasonally affected pixels effectively. In addition, the seasonal transitions were smoother in our proposed relaxation algorithm than in the IR-MAD using a ring network. These results highlight the effectiveness of our approach in handling complex image datasets.

#### 3.1.2. Water and Topographical Features

**Figure 5:**
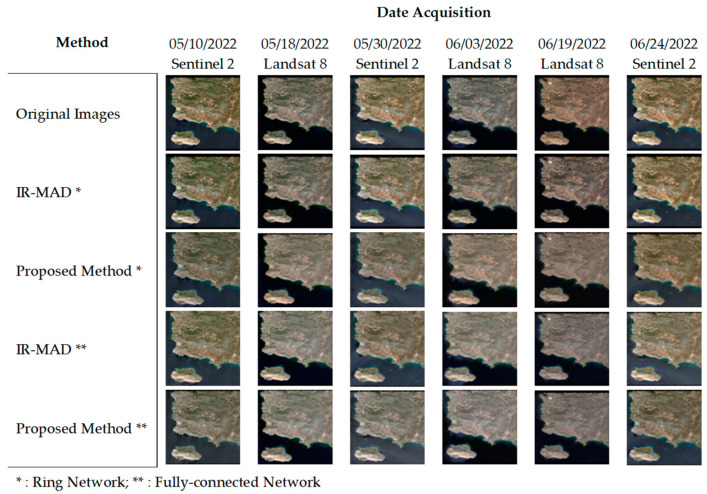
Comparison of normalization results for water- and topographical-features images, Greece image dataset.

Figure 5 presents the Greece image dataset, which focuses on areas with water and topographical features. Overall, normalization was performed effectively on this dataset. However, image inconsistency still occurred. The presence of water pixels could be one of the factors contributing to this inconsistency. Water has a high reflectance in blue and green spectral bands, but low reflectance in red and near-infrared bands, resulting in color variations that can vary significantly, depending on the spectral bands used in the image [48]. Additionally, water surfaces can be affected by changes in lighting conditions and atmospheric effects, which can further complicate the normalization process. Moreover, the effect of topography on normalization can also pose challenges in maintaining consistent image features across different terrain types. In areas with significant topographies, there can be variations in lighting conditions, shading, and perspective, which can affect the appearance of images [49,50]. This can result in losses of contrast and detail in areas with high elevation, or the overemphasis of features in areas with low elevation. Our proposed relaxation algorithm and IR-MAD using a fully connected network may present difficulties in maintaining topographical features during normalization, which can lead to decreased contrast in images.

#### 3.1.3. Desert Features

**Figure 6:**
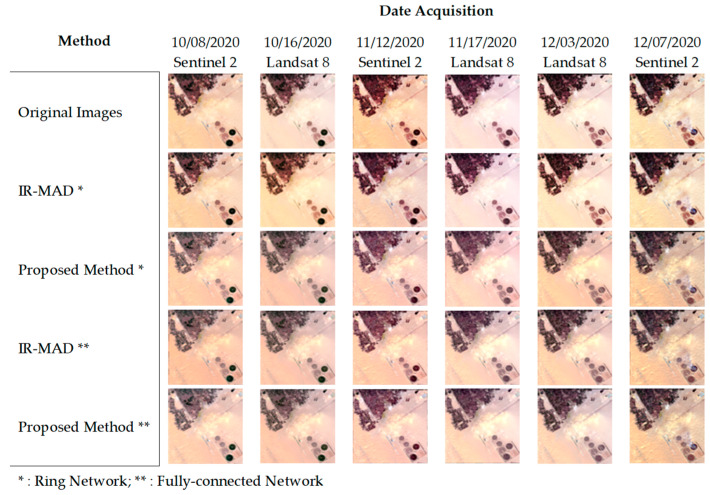
Comparison of normalization results for desert-features images, Egypt image dataset.

Figure 6 presents the Egypt image datasets. From this dataset, we found that our proposed relaxation algorithm with IR-MAD using the fully connected network showed a similar level of improvement in image consistency. However, the improvement using IR-MAD with the ring network was only partial and only applied to some of the images. As shown in the figure, we observed the presence of bright pixels in the middles of the images. These pixels may have been caused by different factors, including sensor noise, saturation, and atmospheric effects [51]. In addition, aerosol effects, such as dust and sand particles, which are common in desert images, may also have contributed to the appearance of these bright pixels [52]. Another possibility is that the normalization process introduced artifacts, which led to the bright pixels.

#### 3.1.4. Snow Features

**Figure 7:**
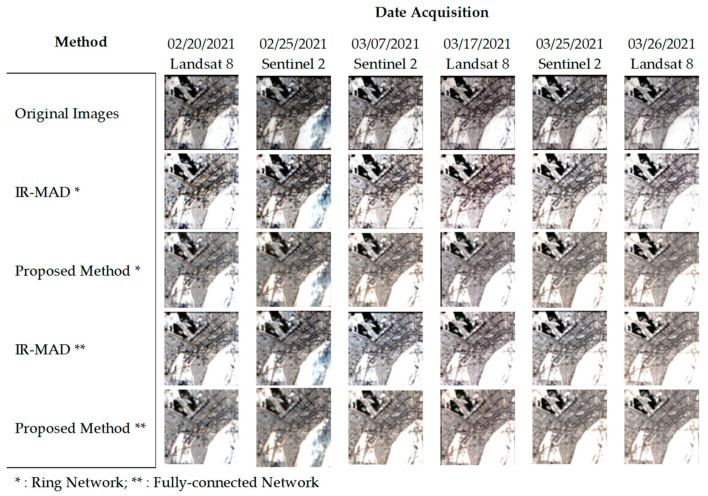
Comparison of normalization results for snow-features images, Russia image dataset.

Figure 7 shows the Russia image dataset, which consisted of snowy images. However, it was difficult to assess the effectiveness of the normalization methods, including IR-MAD and our proposed relaxation algorithm, due to the dominance of snow pixels in the images. Therefore, the normalization methods applied to these images may not have exerted a significant impact on their appearance. This made it difficult to visually assess any improvements in the consistency of the images after the normalization. Nevertheless, we observed a significant change in the second image of the dataset, which became more similar to its neighboring images after the normalization process.

#### 3.1.5. Cloud Features

**Figure 8:**
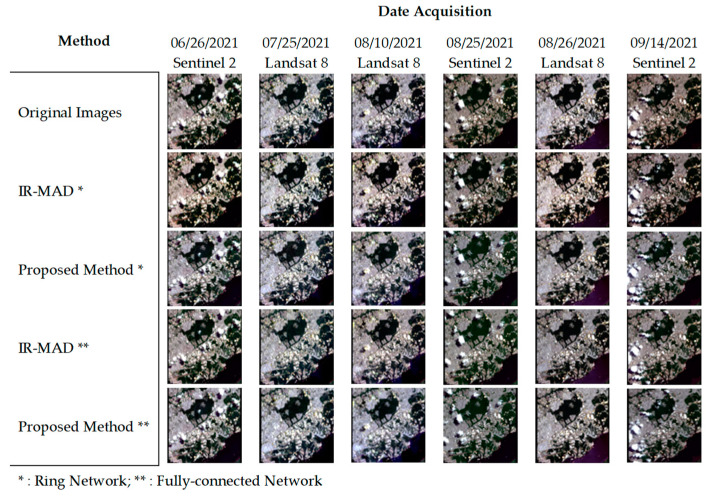
Comparison of normalization results for cloud-feature images, Brazil image dataset.

Figure 8 presents the Brazil image dataset, which focused on urban areas but also included images with cloud presence. We found that despite the presence of clouds, image normalization was still effectively conducted using both IR-MAD and our proposed relaxation algorithm. While the appearance of objects behind the clouds improved after the normalization, the clouds themselves remained relatively unchanged in appearance. However, we did not observe any noticeable artifacts or distortions in the cloud features resulting from the normalization process. Overall, the normalization methods, especially our proposed relaxation algorithm, showed promising results in reducing image inconsistencies and improving visual consistency.

### 3.2. Quantitative Assessment

The quantitative assessment of the image-normalization process involves several methods, including spectral comparison, temporal comparison, and statistical comparison. Spectral comparison involves comparing the reflectance values of different wavelengths to identify differences in spectral response. Temporal comparison involves comparing the surface-reflectance values of the same area over time to identify changes or trends. Statistical comparison involves calculating various statistical parameters, such as the mean and correlation coefficient, to assess the accuracy and consistency of normalization results. Together, these methods provide a comprehensive assessment of the effectiveness of the image-normalization process.

#### 3.2.1. Spectral Comparisons

**Figure 9:**
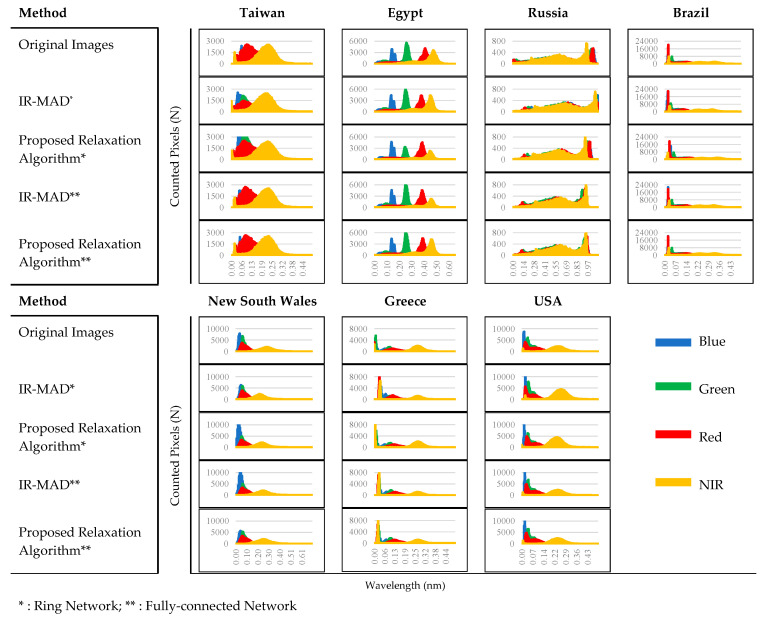
Histogram comparisons of the images before normalization and after normalization.

Spectral comparison involves comparing the histograms of surface-reflectance values of the same image before and after normalization. This comparison provides insights into the distribution of surface-reflectance values across different spectral bands and how they change due to normalization [53]. Significant changes in the shape or distribution of the histogram may indicate problems in the normalization process or highlight differences in the surface-reflectance values of an image before and after normalization. Figure 9 displays histograms for images from each dataset that compare the original images (before normalization) and the IR-MAD and relaxation-algorithm images (after normalization). The histograms of the IR-MAD and proposed relaxation algorithm showed similar distributions to the original images, indicating that both normalization methods preserved the original distribution of the surface-reflectance values in the images. This suggests that both methods can successfully normalize images by maintaining the features and details in images during the normalization process [54].

#### 3.2.2. Temporal Comparisons

**Figure 10:**
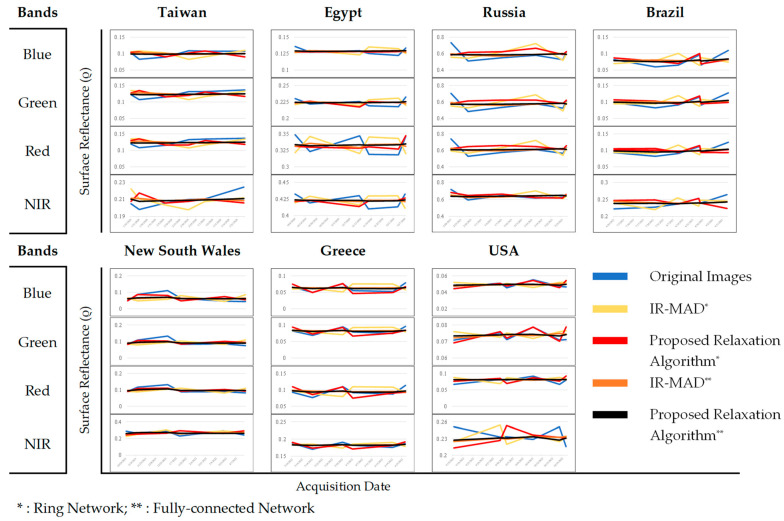
Temporal surface-reflectance comparisons between images before normalization and after normalization.

Temporal comparison involves analyzing changes in surface-reflectance values over time. Figure 10 shows the temporal trend of surface reflectance between the original images, those obtained through IR-MAD, and those from the relaxation algorithm. The surface-reflectance value in the figure is the average value of the surface-reflectance of a given area over a time period. This value was calculated by adding up all the individual reflectance values of each pixel in the area and dividing the total by the number of pixels. Based on Figure 10, it is evident that the design of the network plays a crucial role in improving the consistency of surface-reflectance values between multitemporal cross-sensor images. Our proposed relaxation algorithm and the IR-MAD using a fully connected network aligned well with the original images in that they followed the same trend, with relatively consistent surface-reflectance values over time, as depicted by the black and orange lines in Figure 10. On other hand, the proposed relaxation algorithm and the IR-MAD using a ring network demonstrated a similar pattern, with the IR-MAD presenting a surface-reflectance value that contradicted the original, and our method attempted to reduce the difference but remained opposite to the original. The large disparity between the normalized images may have been due to various factors, such as the complexity of the scene, or the atmospheric conditions.

#### 3.2.3. Loss-Value Measurements

Loss-value measurements assess the effectiveness of a normalization algorithm by evaluating the change in the total error value throughout the iteration process. This measurement helps to determine whether an algorithm achieves a minimum error value. Ideally, the objective value should decrease with each iteration, indicating that the algorithm is making progress towards minimizing the differences between normalized images [43,55].

**Figure 11:**
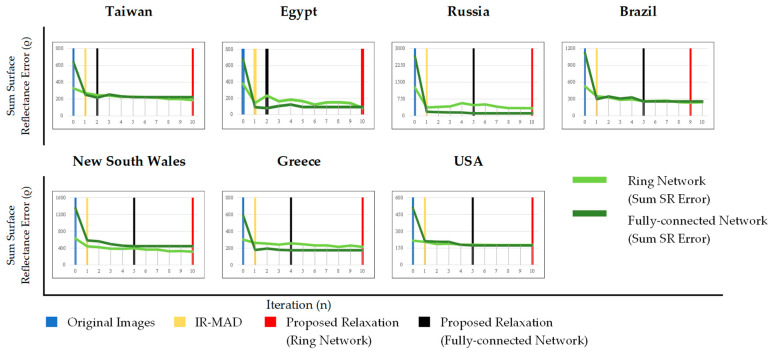
Graph of fluctuations in loss value over iterations.

The fluctuations in the loss-values in this study are presented in Figure 11. The initial objective value in iteration-0 is the original loss-value obtained by Equation (11) for the ring network and Equation (12) for the fully connected network. Iteration-1 represents the IR-MAD’s loss value, while the proposed relaxation’s loss-value is selected from the minimum value of iterations 2 to 10. Based on Figure 11, the overall normalization using the ring network and the fully connected network presented the same trends through the iterations. This figure shows that our proposed relaxation method using a fully connected network needs between one and five additional iterations to achieve a minimum loss-value, while the proposed relaxation algorithm using the ring network needs nine additional iterations. In addition, the normalizations using the ring network had large fluctuations compared with the normalizations using the fully connected networks, which indicates that the normalization using the ring network may have been less efficient.

The specific loss-values in this study are listed in Table 2. According to the table, our proposed relaxations outperformed the IR-MAD algorithm in terms of reducing the error value. In particular, the relaxation algorithm achieved a much lower loss value in all locations compared to the IR-MAD algorithm. This suggests that the relaxation algorithm is more effective in minimizing the differences between normalized images and, thus, in improving the consistency of surface-reflectance values between multitemporal cross-sensor images.

#### 3.2.4. Accuracy Measurements

The accuracy measurement in this study involved evaluating the error between the normalized images using metrics such as the mean absolute error (MAE) and the root mean square error (RMSE). These metrics provide an indication of how close the normalized images are to each other and identify any differences or discrepancies between them [56,57]. Smaller MAE and RMSE values indicate better alignment and similarity between normalized images. The formula of the MAE and RMSE between two normalized images are written in Equations (15) and (16).

(15) $$MAE=\frac{\sum_{i=1}^{N}\left|P_{i}-M_{i}\right|}{N},$$

(16) $$RMSE=\sqrt{\frac{\sum_{i=1}^{N}{\left(P_{i}-M_{i}\right)}^{2}}{N}},$$

where $P_{i}$ is the surface-reflectance value in pixel *i* of the normalized image, $M_{i}$ is the corresponding surface-reflectance value in pixel *i* of the other normalized image, and *N* is the total number of pixels in the image.

Table 3 presents the accuracy measurements from the original images (before normalization), IR-MAD, and proposed relaxation (both after normalization). Based on the table, the MAE and RMSE values for relaxation were consistently lower than those for the IR-MAD and the original images. This suggests that our proposed relaxation algorithm is a more effective normalization method for reducing image discrepancies and errors.

#### 3.2.5. Correlation Measurements

Correlation measurements are used to evaluate the similarity between images before normalization and after normalization. The correlation coefficient ranges from −1 to 1, with 1 indicating a perfect positive correlation, 0 indicating no correlation, and −1 indicating a perfect negative correlation. A correlation coefficient close to 1 suggests that the normalized images are highly similar to the original images, while a correlation coefficient close to 0 indicates a low similarity between the images [58]. In this stage, the correlation coefficient is calculated for each spectral band for the entire image, formulated in Equation (17).

(17) $$\rho \left(P,M\right)=\frac{1}{k}\sum_{j=1}^{k}\frac{\left(P_{j}-{\overset{-}{P}}_{j}\right)}{\sigma P}\frac{\left(M_{j}-{\overset{-}{M}}_{j}\right)}{\sigma M},$$

where *k* represents the number of bands, $P_{j}$ and $M_{j}$ are the surface-reflectance values of the *j*-th band in two images, *P* and *M*, ${\overset{-}{P}}_{j}$ and ${\overset{-}{M}}_{j}$ are the means value of two images, and σP and σM are the standard deviations of *P* and *M*, respectively.

Table 4 shows the correlation measurements for the original images, the IR-MAD, and the relaxation algorithm for the image normalization. According to the table, the IR-MAD achieved the highest correlation coefficient in some of the experiments. However, the overall assessment shows that our proposed relaxation method achieved the highest correlation coefficient among the three methods (original, IR-MAD, and relaxation). This indicates that our relaxation algorithm is able to effectively maintain image similarity across different spectral bands before and after normalization, leading to a more accurate and consistent image representation.

#### 3.2.6. Spectral Distance Measurements

Spectral distance measurements are used to evaluate similarity between normalized images [4]. Generally, there are two methods for distance measurements: Euclidean distance (ED) and spectral angle mapper (SAM). The Euclidean distance is a measure of the distance between two points in a multidimensional space [59]. In the context of image normalization, the points represent the surface-reflectance values of each pixel in the images. A shorter Euclidean distance indicates a greater degree of similarity between two pixels. The Euclidean distance between two pixels is calculated using the following formula:

(18) $$D_{ED}(P,M)=\sqrt{\sum_{i=1}^{N}{\left(P_{i}-M_{i}\right)}^{2}},$$

where $P_{i}$ and $M_{i}$ are the surface reflectance of pixel *i* in the two normalized images, *P* and *M*.

On other hand, the spectral angle mapper is a measure of the angle between the spectral vectors of two pixels [60]. A smaller spectral angle indicates a greater degree of similarity between two pixels. The spectral angle between two pixels is calculated using the following formula:

(19) $$D_{SAM}(P,M)=\cos^{-1}\left(\frac{\sum_{i=1}^{N}P_{i}M_{i}}{\sqrt{\sum_{i=1}^{N}P_{i}^{2}}\sqrt{\sum_{i=1}^{N}M_{i}^{2}}}\right),$$

Table 5 shows the spectral distance measurements of the Euclidean distance and spectral angle mapper for the original images, the IR-MAD images, and the relaxation-algorithm images. The purpose of comparing the spectral distance before and after normalization is to determine improvements in image similarity. In some of the experiments, the IR-MAD achieved better spectral distance measurements. However, the proposed relaxation algorithm outperformed the IR-MAD and the original images, with consistently lower spectral distance values. This suggests that our relaxation algorithm is more effective in reducing the differences between images, resulting in a more similar representation of images after normalization.

Based on the visual assessment, our proposed relaxation algorithm was found to outperform the IR-MAD and the original images in terms of improving the image consistency. Despite the complexity of some of the datasets, including the presence of clouds, water, and seasonal changes, our approach effectively reduced the image inconsistencies while maintaining the important features. However, it was observed that our relaxation algorithm was not able to preserve topographic features as well as the IR-MAD using the ring network. Therefore, the statistical assessment results suggest that our proposed relaxation produced better assessments than the IR-MAD and the original images. There could be several possible reasons behind the superior performance of our proposed relaxation algorithm r.

Firstly, our proposed relaxation algorithm incorporates a looping structure that repeats the normalization process until a desired level of image similarity is achieved. This allows the IR-MAD to continually refine the normalization parameters based on the difference between the current normalized image and its paired image. As a result, the algorithm can adjust the normalization parameters to better match the paired image, leading to a more accurate and consistent normalization. This iterative approach enables the relaxation algorithm to effectively reduce discrepancies between images, resulting in better assessments compared to the original images and the IR-MAD. Moreover, our proposed relaxation can ensure that normalization is applied consistently across all images. This is because the normalization parameters may vary slightly between images. Therefore, the iteration process helps to ensure the normalization is applied consistently to all images, which can improve the overall image similarity.

Based on the experimental results, our proposed relaxation algorithm demonstrated high effectiveness in improving the image consistency for various satellite-image datasets. However, one of its limitations is that it may not preserve certain features in images. For example, in some cases, topographic features may be lost during the normalization process, which can result in a decrease in contrast in the normalized images. Additionally, while our approach was able to maintain seasonal affected pixels, there may still have been some inconsistencies due to the complex features of the dataset, such as clouds and seasonal changes. Furthermore, we found that the improvements in image consistency for snowy images were insignificant. This may have been due to the nature of snow-covered surfaces, which are already uniform in color and texture, making it difficult for normalization methods to exert a significant impact. Therefore, it is important to carefully assess the results of the normalization process and ensure that the key features of the images are preserved while considering the limitations of the relaxation process.

## 4. Conclusions

Based on the findings of this study, the proposed relaxation algorithm demonstrated its effectiveness in enhancing the image consistency in multitemporal cross-sensor-image datasets. The combination of qualitative and quantitative assessments revealed that our algorithm surpassed both the IR-MAD method and the original images in reducing the image inconsistencies, preserving the crucial features, and enhancing the accuracy and consistency of the surface-reflectance values. These results indicate that the relaxation algorithm is a robust solution for addressing the challenges posed by complex datasets, including those with seasonal changes, water, urban areas, and cloud cover.

In addition to the algorithm’s performance, the network design employed in this study deserves attention. Two different network configurations, namely a ring network and a fully connected network, were utilized for the relaxation process in image normalization. The ring network connected each image in the dataset to its neighboring images, while the fully connected network established links between all the images. The use of these network designs allowed the comprehensive alignment of the radiometric conditions across all the images without relying on a reference image.

The comparison between the two network configurations demonstrated the significant influence of the network design on the algorithm’s effectiveness. The normalization process using the fully connected network was faster than that using the ring network. In addition, the fully connected network produced superior normalization results to those obtained with the ring network. This suggests that the fully connected network, as the network design of choice, not only ensures faster processing, but also ensures better normalization outcomes.

However, it is important to acknowledge that our relaxation algorithm exhibits limitations in preserving topographic features compared to the IR-MAD method that utilizes the ring network. To overcome this drawback, future studies could explore potential strategies to combine the strengths of these different methods, aiming to achieve further improvements in normalization outcomes for multitemporal cross-sensor images. By leveraging the advantages of both approaches, researchers can potentially enhance the preservation of topographic features while maintaining the overall effectiveness of the relaxation algorithm.

Overall, this study emphasizes the significance of employing effective image-normalization methods to enhance the accuracy and consistency of multitemporal cross-sensor-image datasets. The proposed relaxation algorithm stands as a valuable tool for researchers and practitioners working with these types of datasets, offering enhanced capabilities for reliable analysis. Moreover, further research could investigate the use of the relaxation algorithm in different remote-sensing applications to assess its potential in diverse contexts, expanding its utility beyond the scope of this study. By exploring its adaptability and performance in various scenarios, we can gain deeper insights into the versatility and broader applications of the relaxation algorithm in the field of remote sensing.

## Figures and Tables

**Table 1:** Multitemporal cross-sensor datasets used in the experiments.

Dataset	Location	Acquisition Date	Geographical Features	Total Image
#1	Tainan, Taiwan	January–March 2020	Clear-sky conditions over urban area and airport	6
#2	Mut, Egypt	October–December 2020	Small village in desert area	6
#3	Yakutsk, Russia	February–March 2021	Snow-covered urban area	6
#4	Manaus, Brazil	June–September 2021	Cloud pixels and urban area	6
#5	Dubbo, New South Wales, Australia	October 2021–April 2022	Cloud pixels and urban area	6
#6	Legrena, Athens, Greece	May–June 2022	Water body, hill, valley, and shadow	6
#7	Nashville, Tennessee, USA	September–October 2022	Seasonally affected urban area	6

**Table 2:** Comparisons of loss-values of original images and normalized images, which represent the total error between the images in each dataset. Bold font indicates the lower value of loss-values.

	Loss-Value Measurments							
Method	Taiwan	Egypt	Russia	Brazil	New South Wales	Greece	USA	Overall
Original Images	327.9	376.0	1259.3	524.3	626.3	301.2	216.0	518.7
Ring Network								
IR-MAD	271.6	141.5	375.2	347.1	444.5	262.8	204.8	292.5
Proposed Relaxation Algorithm	**187.5**	**83.1**	**339.2**	**235.8**	**316.3**	**216.0**	**177.7**	**222.2**
Fully Connected Network								
IR-MAD	250.8	90.8	186.3	296.5	583.1	178.5	211.8	256.8
Proposed Relaxation Algorithm	**217.1**	**79.2**	**116.3**	**257.3**	**446.1**	**175.6**	**173.5**	**209.3**

**Table 3:** Comparisons of accuracy measurements of original images and normalized images by using mean absolute error (MAE) and root mean square error (RMSE). Bold font indicates the lower value of MAE or RMSE.

	**Accuracy Measurements**							
**Method**	**Taiwan**		**Egypt**		**Russia**		**Brazil**	
	**MAE**	**RMSE**	**MAE**	**RMSE**	**MAE**	**RMSE**	**MAE**	**RMSE**
Original Images	7.1	8.5	7.5	9.0	29.3	35.2	12.4	15.7
Ring Network								
IR-MAD	6.3	7.5	5.1	6.0	9.3	11.1	7.6	9.3
Proposed Relaxation Algorithm	**4.3**	**5.2**	**1.7**	**2.1**	**5.8**	**6.9**	**6.2**	**7.6**
Fully Connected Network								
IR-MAD	2.8	3.4	1.0	1.2	2.1	2.5	3.3	4.1
Proposed Relaxation Algorithm	**2.4**	**3.0**	**0.9**	**1.0**	**1.1**	**1.3**	**2.9**	**3.7**
**Method**	**New South Wales**		**Greece**		**USA**		**Overall**	
	**MAE**	**RMSE**	**MAE**	**RMSE**	**MAE**	**RMSE**	**MAE**	**RMSE**
Original Images	14.9	19.4	6.5	7.8	5.6	6.8	11.9	14.6
Ring Network								
IR-MAD	10.1	12.2	5.4	6.3	4.6	5.5	6.9	8.3
Proposed Relaxation Algorithm	**7.6**	**9.3**	**4.3**	**5.0**	**3.1**	**3.7**	**4.7**	**5.7**
Fully Connected Network								
IR-MAD	6.5	8.1	2.0	2.4	2.4	2.9	2.9	3.5
Proposed Relaxation Algorithm	**5.0**	**6.1**	**2.0**	**2.4**	**1.7**	**2.1**	**2.3**	**2.8**

**Table 4:** Comparisons of correlation coefficients of original images and normalized images for each dataset. Bold font indicates the higher value of correlation coefficients.

	Correlation Measurements							
Method	Taiwan	Egypt	Russia	Brazil	New South Wales	Greece	USA	Overall
Original Images	87.86%	94.50%	78.18%	90.06%	63.44%	93.05%	91.12%	85.46%
Ring Network								
IR-MAD	87.88%	**97.98%**	79.88%	92.24%	**64.10%**	93.95%	91.25%	86.75%
Proposed Relaxation Algorithm	**87.90%**	97.64%	**82.23%**	**92.45%**	63.47%	**94.70%**	**91.25%**	**87.09%**
Fully Connected Network								
IR-MAD	87.86%	97.72%	79.56%	**94.16%**	64.10%	**94.32%**	**90.88%**	86.94%
Proposed Relaxation Algorithm	**88.24%**	**98.06%**	**84.13%**	93.62%	**64.43%**	94.18%	90.29%	**87.56%**

**Table 5:** Similarity assessment by using spectral distance measurements of original images and normalized images. Bold font indicates the lower value of spectral distance in Euclidean Distance (ED) and Spectral Angle Mapper (SAM).

	**Spectral Distance Measurements**							
**Method**	**Taiwan**		**Egypt**		**Russia**		**Brazil**	
	**ED**	**SAM**	**ED**	**SAM**	**ED**	**SAM**	**ED**	**SAM**
Original Images	5.96	26.17	6.09	16.02	22.60	30.50	9.50	30.40
Ring Network								
IR-MAD	4.73	24.14	4.35	13.24	7.24	**10.85**	6.27	**26.10**
Proposed Relaxation Algorithm	**3.99**	**22.79**	**1.99**	**7.73**	**6.28**	11.65	**5.63**	26.72
Fully Connected Network								
IR-MAD	2.29	15.67	0.95	4.56	1.72	3.31	**2.37**	**16.03**
Proposed Relaxation Algorithm	**2.14**	**15.44**	**0.72**	**3.93**	**0.78**	**1.82**	3.02	17.85
**Method**	**New South Wales**		**Greece**		**USA**		**Overall**	
	**ED**	**SAM**	**ED**	**SAM**	**ED**	**SAM**	**ED**	**SAM**
Original Images	12.19	33.35	5.02	36.51	4.91	27.51	9.47	28.64
Ring Network								
IR-MAD	8.76	29.65	4.31	33.10	3.79	25.55	5.64	23.23
Proposed Relaxation Algorithm	**7.15**	**26.19**	**3.52**	**28.61**	**3.63**	**23.22**	**4.60**	**20.99**
Fully Connected Network								
IR-MAD	5.01	21.26	**1.45**	17.84	**1.84**	14.88	2.23	13.36
Proposed Relaxation Algorithm	**4.51**	**18.35**	1.61	**17.11**	1.96	**13.70**	**2.11**	**12.60**

ED: Euclidean distance; SAM: spectral angle mapper.

**Figure A1:**
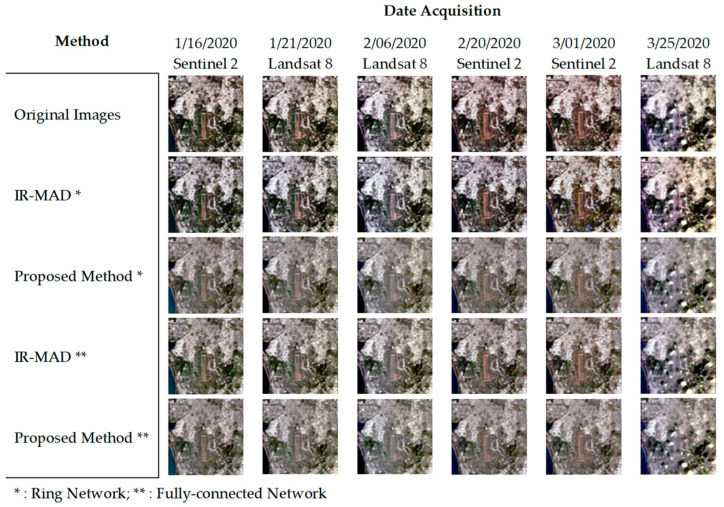
Comparison of normalization results for urban-area images, Taiwan image datasets.

**Figure A2:**
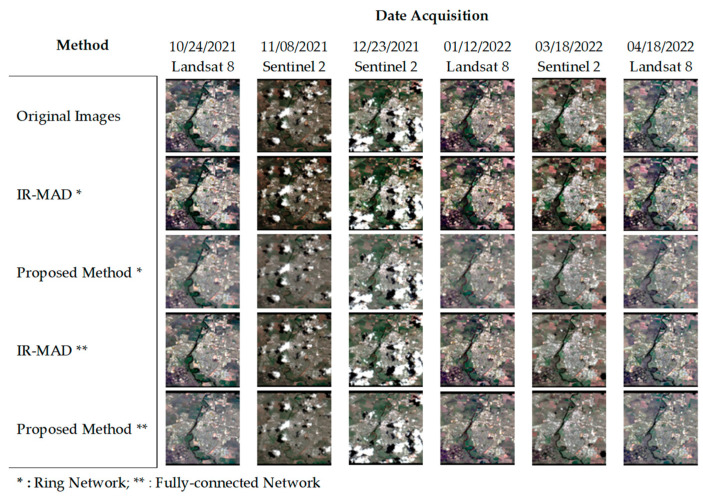
Comparison of normalization results for cloud-feature and seasonal images, Australia image datasets.

## Data Availability

Not applicable.

## Appendix A

The following figures (Figure A1 and Figure A2) present the results of the normalization of the urban-features images in Taiwan and the cloud and seasonal features in Australia, respectively, using the proposed relaxation algorithm and the IR-MAD algorithm with fully connected and ring networks. A visual comparison of the figures reveals that the proposed relaxation algorithm produced normalized images that were visually comparable to those obtained using the IR-MAD algorithm with the fully connected networks. However, the normalized images from the IR-MAD algorithm with the ring network show minor changes compared to the original images, indicating that the inconsistency was not effectively reduced. Therefore, the proposed relaxation algorithm outperformed the IR-MAD algorithm in terms of reducing the image inconsistency.
